# Supplementary material for: Sweet chestnut standardized fractions from sustainable circular process and green tea extract: In vitro inhibitory activity against phytopathogenic fungi for innovative applications in green agriculture
Source: PLoS One. 2021 Feb 22;16(2):e0247298. doi: 10.1371/journal.pone.0247298 (PMC7899350; doi:10.1371/journal.pone.0247298)
Supplement: Attachment — Submitted filename: Response to Reviewers.pdf [file pone.0247298.s004.pdf]

Florence, January 19th, 2021

Dear PLOS ONE Editors and Reviewers,

All the authors would like to thank the Editor and Reviewers for their careful reading of the manuscript PONE-D-20-10008R1 entitled “Sweet chestnut standardized fractions from sustainable circular process and green tea extract: *in vitro* inhibitory activity against phytopathogenic fungi for innovative applications in green agriculture”.

The manuscript has been revised according to all Reviewers’ comments and all corrections have been marked-up in the copy labelled “Revised Manuscript with Track Changes”; as requested, also an unmarked version without tracked changes, labelled “Manuscript”, has been uploaded.

Comment 1:

Author should consider using either common name or scientific name in the title and use both on the first mention in the manuscript. Then choose which to use throughout and make it consistent.

For sweet chestnut and green tea, only common names were kept in the title; on the first mention both in the Abstract and in the text (Introduction section), common names with the complete scientific names are reported; only common names are used for the following mentions. Also pathogens names were checked and fixed.

Abstract: Line 39-40: “can attack also plants in the field” can be stated as “can also attack field plants”.

Done.

Comment 2:

Line 83: Should include the following reference:

Mohammed Bule *et al.* Tannins (hydrolysable tannins, condensed tannins, phlorotannins, flavono-ellagitannins). Recent Advances in Natural Products Analysis. Elsevier, 2020, pp. 132-146.

Though the introduction is relevant and theory based, sufficient information on previous findings is not presented well for the readers to follow the present study rationale.

The reference was added in the text.

The “Introduction” section has been revised and improved (lines 156-167 in particular) in order to let the readers better understand our study rationale.

Comment 3:

In method section: Lines 167-173: Author may consider including the flow diagram of the dry extraction process.

The flow diagram of the extraction and fractionation industrial process was already reported in a previous publication (Campo et al., 2016), so it is no more possible to use the figure. Here we cited the articles where we described accurately the whole process also by showing the flow diagram (Campo et al., 2016; Lucarini et al., 2018).

Comment 4:

Line 226-227: Must provide the reference to “previously described”.

As it could seem like a reference to literature, in the place of “as previously described”, we have inserted “as described above (see “*In vitro* test of radial growth inhibition”)”. The reference, indeed, is to what described in the previous paragraph.

Comment 5:

Line 266: Consider using “Campo et al.” instead of “this research group”.

“Campo et al.” was added instead of “this research group”.

Comment 6:

Line 275: Please clarify “6-months and 12-months” or “12-months”.

In the place of “The dried SCW fraction was chemically stable at the 6-months and 12-months as demonstrated by the analytical controls”, we added the following sentence: “The dried SCW fraction was chemically stable as demonstrated by control HPLC/DAD analysis repeated after 6 and 12 months.”. This new sentence should be more clear than the first one.

Comment 7:

Consider proofreading the manuscript.

The whole manuscript has been carefully revised and checked. Some typos were fixed and some sentences were re-written to make them more clear. Particular attention was paid to the English language.
