# Supplementary material for: Sweet chestnut standardized fractions from sustainable circular process and green tea extract: In vitro inhibitory activity against phytopathogenic fungi for innovative applications in green agriculture
Source: PLoS One. 2021 Feb 22;16(2):e0247298. doi: 10.1371/journal.pone.0247298 (PMC7899350; doi:10.1371/journal.pone.0247298)
Supplement: Attachment — Submitted filename: Review Report_PLOS ONE - 16.6.2020.docx [file pone.0247298.s001.docx]

Dear Editor,

**Reviewer’s Report: PONE-D-20-10008**

Sweet chestnut (Castanea sativa Mill.) standardized fractions from sustainable circular process and green tea (Camellia sinensis L.) extract: in vitro inhibitory activity against phytopathogenic fungi for innovative applications in green agriculture

**Here are some minor corrections:**

Line 316-318 figures title should be removed

Line 353 space should be removed

I suggest to **Accept** the manuscript for publication in PLOS ONE.
