# Supplementary material for: Sweet chestnut standardized fractions from sustainable circular process and green tea extract: In vitro inhibitory activity against phytopathogenic fungi for innovative applications in green agriculture
Source: PLoS One. 2021 Feb 22;16(2):e0247298. doi: 10.1371/journal.pone.0247298 (PMC7899350; doi:10.1371/journal.pone.0247298)
Supplement: Attachment — Submitted filename: Response to Reviewers.doc [file pone.0247298.s002.doc]

1. Please ensure that your manuscript meets PLOS ONE's style requirements, including those for file naming. The PLOS ONE style templates can be found at

https://journals.plos.org/plosone/s/file?id=wjVg/PLOSOne_formatting_sample_main_body.pdf and

<https://journals.plos.org/plosone/s/file?id=ba62/PLOSOne_formatting_sample_title_authors_affiliations.pdf>

We would like to thank the Editors for their comments. We checked PLOS ONE style templates and ensured that our manuscript meets the style requirements.

2.We suggest you thoroughly copyedit your manuscript for language usage, spelling, and grammar. If you do not know anyone who can help you do this, you may wish to consider employing a professional scientific editing service.

We had the manuscript checked for language usage, spelling, and grammar and fixed some inaccuracies, as evidenced in the text of our Manuscript.

3.Thank you for stating the following in the Financial Disclosure section:

[This research is part of the following projects:  Tuscany Region, POR FESR 2014/2020. Nat-BackeryInnov "Innovative production of a bakery line based on natural functional extracts for wellness and sports”; Tuscany Region, POR CREO FESR 2014-2020 “Formulation of new sustainable products for agriculture based on natural extracts”, LIFE18 ENV/NL/000043 - LIFE “PlantsforPlants”.].

We note that one or more of the authors are employed by a commercial company: PIN scrl

1. Please provide an amended Funding Statement declaring this commercial affiliation, as well as a statement regarding the Role of Funders in your study. If the funding organization did not play a role in the study design, data collection and analysis, decision to publish, or preparation of the manuscript and only provided financial support in the form of authors' salaries and/or research materials, please review your statements relating to the author contributions, and ensure you have specifically and accurately indicated the role(s) that these authors had in your study. You can update author roles in the Author Contributions section of the online submission form.

Please also include the following statement within your amended Funding Statement.

“The funder provided support in the form of salaries for authors [insert relevant initials], but did not have any additional role in the study design, data collection and analysis, decision to publish, or preparation of the manuscript. The specific roles of these authors are articulated in the ‘author contributions’ section.”

If your commercial affiliation did play a role in your study, please state and explain this role within your updated Funding Statement.

2. Please also provide an updated Competing Interests Statement declaring this commercial affiliation along with any other relevant declarations relating to employment, consultancy, patents, products in development, or marketed products, etc.

Within your Competing Interests Statement, please confirm that this commercial affiliation does not alter your adherence to all PLOS ONE policies on sharing data and materials by including the following statement: "This does not alter our adherence to  PLOS ONE policies on sharing data and materials.” (as detailed online in our guide for authors [http://journals.plos.org/plosone/s/competing-interests](about:blank)) . If this adherence statement is not accurate and  there are restrictions on sharing of data and/or materials, please state these. Please note that we cannot proceed with consideration of your article until this information has been declared.

Please include both an updated Funding Statement and Competing Interests Statement in your cover letter. We will change the online submission form on your behalf.

Please know it is PLOS ONE policy for corresponding authors to declare, on behalf of all authors, all potential competing interests for the purposes of transparency. PLOS defines a competing interest as anything that interferes with, or could reasonably be perceived as interfering with, the full and objective presentation, peer review, editorial decision-making, or publication of research or non-research articles submitted to one of the journals. Competing interests can be financial or non-financial, professional, or personal. Competing interests can arise in relationship to an organization or another person. Please follow this link to our website for more details on competing interests: [http://journals.plos.org/plosone/s/competing-interests](about:blank)

We would like to thank the Editors for pointing this. The affiliation reported in the manuscript has been completed by adding a lacking part of the description. PIN scrl is not a commercial company, but an University Center for educational and scientific services for the University of Florence, as now indicated (see the affiliations in the Title page). So the funder of this research is not PIN scrl, but Tuscany Region for projects POR FESR 2014/2020 Nat-BackeryInnov "Innovative production of a bakery line based on natural functional extracts for wellness and sports” and POR CREO FESR 2014-2020 “Formulation of new sustainable products for agriculture based on natural extracts”, and the European Community for LIFE Project Plants for Plants, “Boost conventional agriculture’s confidence: new organic bio-stimulants to reduce water, nutrients and pesticide demand project”, LIFE18 ENV/NL/000043. So we can exclude the existence of competing interests.

Reviewer #1: This paper needs to be carefully reorganized with a clearly stated argument spelled out in the opening pages. The authors should work harder on the approach adopted, establish a clear theoretical background to contextualize the analysis and narrow the scope of the analysis to specific aspects. Apart from compiling key studies, a critical approach to the literature is required. There is a need of offering detailed insights regarding the main purpose of the paper and its contribution. The discussions require more structure and there is a need of offering a clear assessment of reviewed literature. The main contributions of the paper should be presented as part of the empirical discussions or critical assessment on the core research outcomes. The main contribution should be emphasised more and the concluding statements should be stronger. The paper presents its points in a rather descriptive manner, referring to relevant sources but without really discussing them. It brings up interesting points, but stating them rather than arguing for them. The research idea is not properly contextualised, as there is a need of offering a detailed review of relevant literature that help the authors developing the key arguments that support their proposed research. Data gathering and data analysis can be reconsidered and discussed more comprehensively. The lead up argument for the study and results section appear to be too loosely constructed. There is some discussion of the limitations of the study however these are not considered in terms of the implications on the study findings. Some bibliographic references are simply brought up without being developed, or without an adequate explanation as to why they are relevant.

We would like to thank the reviewer for his/her comment. As he/she suggested, the paper has been reorganized, in particular the “Introduction” and “Results and Discussion” sections. Both the results and literature were discussed more in depth, also by adding some new references to recent studies, to better contextualize our study and explain the main purpose and contribution of the paper.

Reviewer #2: Reviewer’s Report: PONE-D-20-10008

Line 316-318 figures title should be removed: we would like to thank the reviewer for his/her comment. According to PlosOne Manuscript body formatting guidelines, “Each figure caption should appear directly after the paragraph in which they are first cited”. For this reason we have kept the figure captions in the same position.

Line 353 space should be removed: the space has been removed.

- Furthermore, although no requests were made, we have replaced the file of Figure 1 by uploading a new one of better quality, as we noticed that the previous image had an insufficient quality.
