# Supplementary material for: Sweet chestnut standardized fractions from sustainable circular process and green tea extract: In vitro inhibitory activity against phytopathogenic fungi for innovative applications in green agriculture
Source: PLoS One. 2021 Feb 22;16(2):e0247298. doi: 10.1371/journal.pone.0247298 (PMC7899350; doi:10.1371/journal.pone.0247298)
Supplement: Attachment — Submitted filename: Comments_PLoS ONE 2.docx [file pone.0247298.s003.docx]

**Comments:**

This manuscript makes a reasonable contribution insofar as it empirically demonstrates the potential antifungal activity of sweet chestnut and green tea towards phytopathogenic fungi and leverage to its application in green agriculture.

However, before the manuscript can be considered for publication, a number of issues would need to be addressed. There are also few smaller inaccuracies that need to be rectified.

**Comment 1:**

Author should consider using either common name or scientific name in the title and use both on the first mention in the manuscript. Then choose which to use throughout and make it consistent.

Abstract: Line 39-40: “can attack also plants in the field” can be stated as “can also attack field plants”

**Comment 2:**

Line 83: Should include the following reference:

Mohammed Bule *et al.* Tannins (hydrolysable tannins, condensed tannins, phlorotannins, flavono-ellagitannins). Recent Advances in Natural Products Analysis. Elsevier, 2020, pp. 132-146.

Though the introduction is relevant and theory based, sufficient information on previous findings is not presented well for the readers to follow the present study rationale.

**Comment 3:**

In method section: Lines 167-173: Author may consider including the flow diagram of the dry extraction process.

**Comment 4:**

Line 226-227: Must provide the reference to “previously described”.

**Comment 5:**

Line 266: Consider using “Campo et al.” instead of “this research group”

**Comment 6:**

Line 275: Please clarify “6-months and 12-months” or “12-months”

**Comment 7:**

Consider proofreading the manuscript.
